# Monitoring Phenotype Heterogeneity at the Single-Cell Level within *Bacillus* Populations Producing Poly-3-hydroxybutyrate by Label-Free Super-resolution Infrared Imaging

**DOI:** 10.1021/acs.analchem.3c03595

**Published:** 2023-11-24

**Authors:** Cassio Lima, Howbeer Muhamadali, Royston Goodacre

**Affiliations:** Centre for Metabolomics Research, Department of Biochemistry, Cell and Systems Biology, Institute of Systems, Molecular and Integrative Biology, University of Liverpool, Liverpool L69 7ZB, U.K.

## Abstract

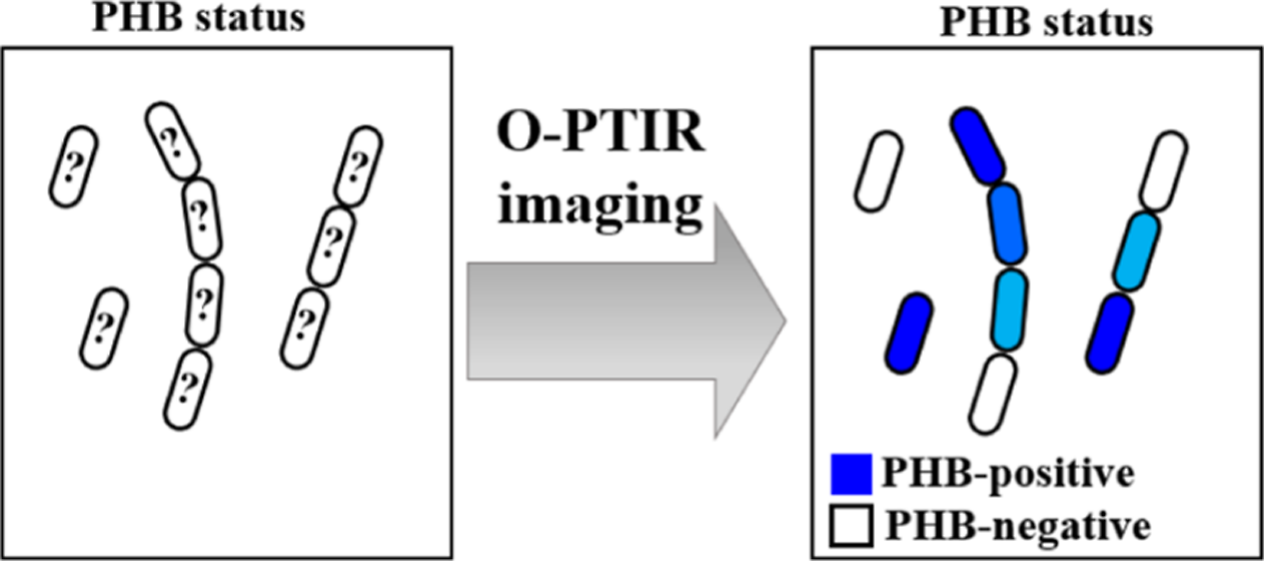

Phenotypic heterogeneity is commonly found among bacterial cells within microbial populations due to intrinsic factors as well as equipping the organisms to respond to external perturbations. The emergence of phenotypic heterogeneity in bacterial populations, particularly in the context of using these bacteria as microbial cell factories, is a major concern for industrial bioprocessing applications. This is due to the potential impact on overall productivity by allowing the growth of subpopulations consisting of inefficient producer cells. Monitoring the spread of phenotypes across bacterial cells within the same population at the single-cell level is key to the development of robust, high-yield bioprocesses. Here, we discuss the novel development of optical photothermal infrared (O-PTIR) spectroscopy to probe phenotypic heterogeneity within *Bacillus* strains by monitoring the production of the bioplastic poly-3-hydroxybutyrate (PHB) at the single-cell level. Measurements obtained on single-point and in imaging mode show significant variability in the PHB content within bacterial cells, ranging from whether or not a cell produces PHB to variations in the intragranular biochemistry of PHB within bacterial cells. Our results show the ability of O-PTIR spectroscopy to probe PHB production at the single-cell level in a rapid, label-free, and semiquantitative manner. These findings highlight the potential of O-PTIR spectroscopy in single-cell microbial metabolomics as a whole-organism fingerprinting tool that can be used to monitor the dynamic of bacterial populations as well as for understanding their mechanisms for dealing with environmental stress, which is crucial for metabolic engineering research.

## Introduction

In recent decades, the production of plastics has rapidly increased due to the high demand for plastic products used by multiple industries in the manufacture of items that have become indispensable in daily life.^1,2^ Most plastics are synthetic polymers derived from petroleum-based sources, which are less susceptible to biodegradation due to their high-molecular weight, hydrophobicity, and cross-linked chemical structure.^3,4^ The low recycling rate of plastic products combined with improper disposal has made plastic pollution a serious threat to the environment.^5,6^ Biopolymers represent a viable alternative to replace synthetic polymers as they can be easily degraded by the action of enzymes found within living organisms such as bacteria, yeast, and fungi, with the final products being carbon dioxide (CO_2_), water, and biomass.^7^ Poly-3-hydroxybutyrate (PHB) is a bioplastic that has gained a lot of attention in the past decades as a potential candidate to replace polypropylene and polyethylene due to its good thermoplastic processability and high-quality mechanical properties.^8,9^

Currently, PHB production relies mainly on the fermentation of organic carbon sources by microbial agents such as bacteria, which synthesizes PHB as a protective carbon store in response to environmental stress, in particular thermal and oxidative stress where the products of PHB also confer resistance.^10^ In the last decades, the effects of growth conditions in PHB biosynthesis have been investigated in order to optimize the total amount of biopolymer produced in a bioreactor as well as the optimal time-point to harvest PHB.^11^ As with most microbiological investigations, most of these studies have been supported by experimental evidence acquired from bulk populations, while little attention has been paid to the impacts of environmental changes to PHB metabolism at the single-cell level and, ultimately, how the overall productivity of bioprocesses is affected by the individual behavior of microbial cell factories.

At the single-cell level, microbial populations display tremendous cell-to-cell variations in phenotypic traits due to random stochastic fluctuations in biochemical processes as well as use these adaptive strategies for population growth and survival.^12−14^ The development of phenotypic heterogeneity within microbial populations may occur due to intrinsic factors such as cell-growth phase and variable gene expression as well as extrinsic external factors such as heat, aeration, or acidity, which cause significant environmental perturbations.^12,15,16^ Metabolic heterogeneity is a major issue in the context of industrial bioprocessing as it may negatively affect the overall productivity;^17^ therefore, monitoring phenotypic instability at the single-cell level is crucial to the development of robust and high-yield bioprocesses.^12,17−19^ Flow cytometry is the most common tool to probe microbial phenotypic heterogeneity in bioprocesses, and it has previously been used to quantify PHB.^18−22^ Advanced imaging methods such as transmission electron microscopy (TEM) and fluorescence-based microscopy are often employed to analyze the formation of PHB granules within cells.^23−27^ Recently, optical photothermal infrared (O-PTIR) spectroscopy has emerged as a tool for single-cell microbial metabolomics with potential applications for monitoring bacterial phenotype heterogeneity including intracellular PHB content.^28−31^ In contrast to TEM, O-PTIR signatures enable biochemical analysis by measuring the infrared spectrum of a single bacterium, which provides important molecular information on small molecules, intermediates, and products of microbial metabolism. Compared to fluorescence-based methods, O-PTIR is a label-free technique and therefore does not rely on commercially available fluorescent probes that are susceptible to photobleaching or may even interfere with cell metabolism.^32^ Here, we discuss the use of O-PTIR spectroscopy to probe phenotypic heterogeneity within microbial populations producing PHB at the single-cell level. First, O-PTIR signatures collected on single-point mode from intact single-cells were compared to infrared spectra acquired from bulk populations through conventional Fourier transform infrared (FTIR) spectroscopy, in order to show the ability of O-PTIR to probe phenotypic heterogeneities within a range of *Bacillus* species and strains. Ultimately, we use the O-PTIR chemical maps obtained in imaging mode to evaluate the spatial distribution and arrangements of PHB-producing cells within microbial populations.

## Experimental Section

### FTIR Spectroscopy

Bulk samples from four biological repeats were prepared by spotting 20 μL aliquots from each of the samples onto a 96-well silicon plate (Bruker Ltd., Coventry, UK). Samples were gently heated to dryness (30 min) at 55 °C using an oven. FTIR analysis was carried out in transmission mode using a Bruker INVENIO infrared spectrometer equipped with an accessory for high-throughput measurements (HTS-XT) and deuterated l-alanine-doped triglycene sulfate detector (DLaTGS). Each spectrum represents the average signal obtained from a 3 mm area in the sample. Spectral data were collected in the mid-IR range (4000–600 cm^–1^) with 64 spectral coadds and 4 cm^–1^ spectral resolution. A total of 16 FTIR spectra were collected from each strain, which included four analytical replicates (four biological repeats × four analytical preparations).

### O-PTIR Spectroscopy

Samples were diluted in deionized water (1:1000), and 2 μL aliquots were transferred to CaF_2_ slides. All samples were dried in the oven prior to analysis. O-PTIR measurements were collected from individual cells using a mIRage infrared microscope (Photothermal Spectroscopy Corp., Santa Barbara, USA), with the pump beam consisting of a tunable four-stage quantum cascade laser (QCL) device, while a continuous wave (CW) 532 nm laser was used to probe the photothermal expansion induced by the pump beam. Spectral data were collected in reflection mode using a Cassegrain 40× objective (0.78 NA). Five single-point spectra were acquired across each bacterial cell over the spectral region of 950–1800 cm^–1^, with 2 cm^–1^ spectral resolution and 10 scans per spectrum, and the average spectrum per cell was considered as representative. Chemical maps were collected on an imaging mode (500 nm step size) by tuning the QCL to wavenumbers associated with amide I (1656 cm^–1^) and the ester carbonyl groups (1740 cm^–1^).

## Results and Discussion

### Single-Point Infrared Spectral Measurements

**Figure 1:**
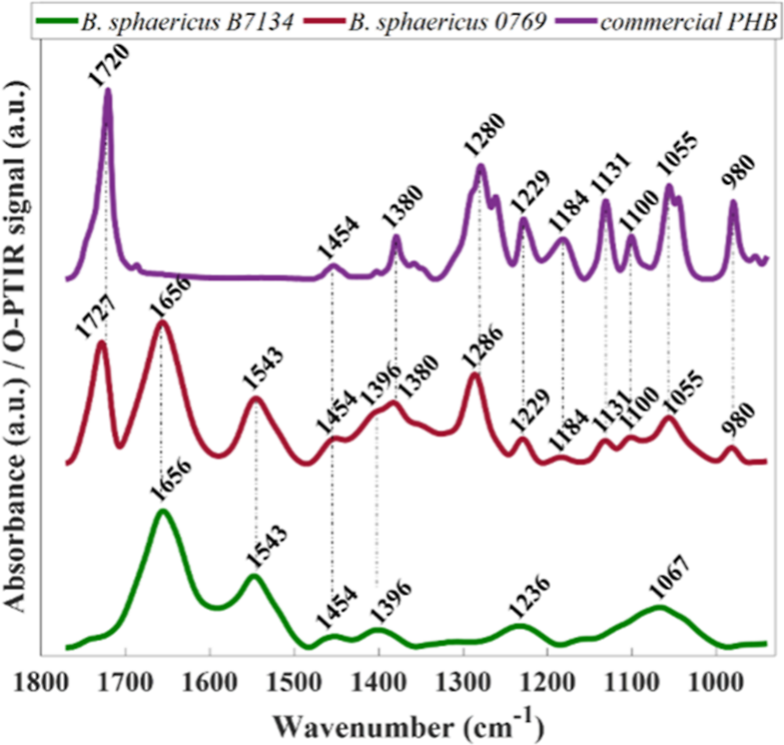
FTIR spectra in the fingerprint region (1800–950 cm^–1^) from commercial PHB (purple, top) as well as of O-PTIR spectra acquired from individual intact cells from PHB-positive (red line, *B. sphaericus* B0769) and PHB-negative (green line, *B. sphaericus* B7134) microbial populations. Plots are offset for clarity, and the main vibrational features are shown.

PHB is a polymer of the polyhydroxyalkanoate (PHA) family that is produced by many microorganisms upon nutrient limitation.^10^ PHB is usually produced by bacteria when there is carbon excess in the environment and low levels of other nutrients such as nitrogen, phosphate, or oxygen, and there is evidence that PHB and its degradation products are protective against environmental stressors.^10^ PHB is found as granules in the bacterial cell cytoplasm.^33^Figure 1 illustrates the fingerprint wavenumber region (1800–950 cm^–1^) of O-PTIR spectra acquired from individual intact cells from PHB-positive (red line, *B. sphaericus* B0769) and PHB-negative (green line, *B. sphaericus* B7134) microbial populations.

The infrared spectrum recorded from a biological sample represents the combination of the infrared signatures from the individual biochemical constituents of the sample. In such situations, the overlapping of bands from different biochemical components may result in the masking of bands with a poor signal. Infrared spectrum collected from PHB-negative bacterium exhibited bands related to chemical vibrations associated with the four major classes of biological macromolecules: proteins (1656, 1543, and 1396 cm^–1^), lipids (1454 and 1396 cm^–1^), nucleic acids (1236 cm^–1^), and carbohydrates (1067 cm^–1^).^30^

Although other bacterial metabolites may be infrared active, their infrared signatures have lower signal compared to the macromolecules previously mentioned and thus, on visible assessment, do not contribute to the overall signatures recorded from a single bacterium.^30^ By contrast, the infrared spectrum obtained from a single bacterium producing PHB is drastically impacted by the PHB signatures, which is clearly a reflection of the level of intracellular PHB. Bands associated with vibrational modes from proteins (1656, 1543, and 1396 cm^–1^) and lipids (1454 and 1396 cm^–1^) can still be seen in the infrared spectrum recorded from PHB-positive bacterium (Figure 1, red line), whereas bands related to nucleic acids and carbohydrates were masked by bands belonging to PHB between 1400 and 950 cm^–1^ (1380, 1286, 1229, 1184, 1131, 1100, 1055, and 980 cm^–1^).^33^ Infrared signatures obtained through FTIR spectroscopy from commercial PHB are also displayed in Figure 1 (purple line) for comparison, which confirms these findings.

The strong band peaking at 1727 cm^–1^ in the infrared signatures of PHB-producing cell is assigned to the ester carbonyl band stretching vibration from PHB—the generalized formulas for this polymer is H–[–O–CH(CH_3_)–CH_2_–C(C=O)–]*n*–OH.^34,35^ A comparison of the infrared signatures from intracellular PHB to PHB extracted from bacteria (commercial PHB) reveals changes in the peak position and intensity of the ester carbonyl band. These findings were also documented by other studies evaluating the PHB content in bulk populations and are attributed to variations in the PHB concentration and crystallinity.^33,36^ The bands peaking between 1400 and 950 cm^–1^ in the PHB spectrum are assigned to CH_3_, CH_2_, and C–O–C functional groups.^33^

**Figure 2:**
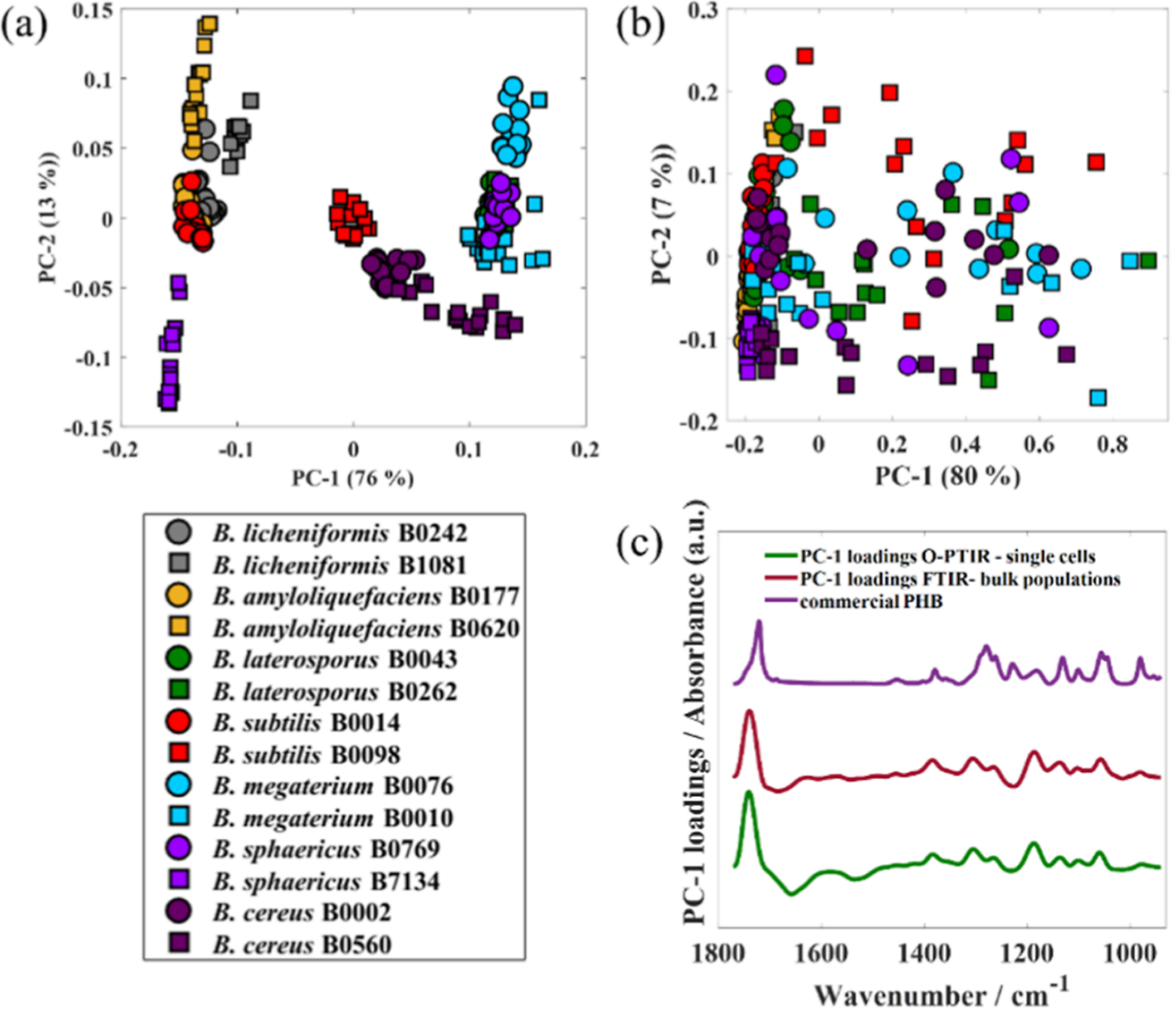
Comparison of the ability of FTIR and O-PTIR to probe the metabolic heterogeneity within *Bacillus* strains producing PHB. (a) Score plot calculated from FTIR spectra acquired from bulk populations. (b) Score plot obtained from the O-PTIR signatures measured from individual bacterial cells; values in parentheses are the percentage of the total explained variance (TEV). (c) PC-1 loading plots from the O-PTIR (green) and FTIR (red) as well as the FTIR spectrum from commercial PHB (green, top). Plots are offset for clarity.

O-PTIR spectra acquired from individual bacterial cells and FTIR spectra collected from bulk populations were subjected to principal component analysis (PCA) in order to explore the ability of O-PTIR spectroscopy to probe variations in the PHB content within PHB-positive and PHB-negative microbial populations. PCA is an unsupervised multivariate statistical method used to reduce the data set dimensionality by creating new variables (i.e., principal components, PCs) based on the original variables.^37^ Besides data set dimensionality reduction, PCA outputs (i.e., scores and loadings plots) are commonly employed to study clustering patterns within data sets. Figure 2a,b illustrates PCA score plots obtained from FTIR and O-PTIR spectral data, respectively. In Figure 2a, scores from *B. sphaericus* B0769 and the two strains of *B. cereus*, *B. megaterium*, and *B. laterosporus* were grouped on the positive axis of PC-1, while scores from *B. subtilis* B0014, *B. sphaericus* B7134, and both *B. licheniformis* and *B. amyloliquefaciens* strains were clustered on the negative axis of PC-1. PCA scores from *B. subtilis* B0098 grouped in both the positive and negative axes of PC-1. High data reproducibility was observed in PCA scores from all bacterial strains; i.e., the scores from spectral data acquired from the same strain grouped close together. Similar to the findings obtained for FTIR data, PCA scores obtained from the O-PTIR spectra acquired from single bacterial cells (Figure 2b) from *B. subtilis* B0014, *B. sphaericus* B7134, and both strains of *B. licheniformis* and *B. amyloliquefaciens* grouped on the negative axis of PC-1. By contrast, PCA scores from the remaining strains (*B. sphaericus* B0769, *B. subtilis* B0098, and the two strains of *B. cereus*, *B. megaterium*, and *B. laterosporus*) can be seen to be located throughout both negative and positive sides of the PC-1 axis with much lower data reproducibility. The loading plots (Figure 2c) were assessed in order to interpret the clustering patterns displayed in Figure 2a,b.

Similar findings were obtained by comparing PC-1 loadings retrieved from FTIR spectra acquired from bulk populations and O-PTIR data collected from single bacterial cells. In both cases, PC-1 loadings are dominated by infrared signatures associated with PHB, indicating that the clustering patterns displayed in the score plots (Figure 2a,b) are due to variations in the cellular PHB content. PCA scores clustered on the PC-1 positive axis relate to infrared spectra of bacterial cells with a higher PHB content, while scores grouped on the negative axis represent spectra of cells with no PHB. According to the PCA scores arrangement, we conclude that the strains *B. sphaericus* B0769, *B. subtilis* B0098, and the two strains of *B. cereus*, *B. megaterium*, and *B. laterosporus* are PHB-producing strains, while the remaining strains (*B. subtilis* B0014, *B. sphaericus* B7134, and both *B. licheniformis* and *B. amyloliquefaciens* strains) are PHB-negative. The perceived poor data reproducibility observed in the PCA score plots calculated from O-PTIR data sets acquired from individual bacterial cells within PHB-producing strains (Figure 2b) is associated with varying intracellular PHB content, and this reflects phenotypic variability at the single-cell level. Intracellular PHB can be accumulated up to 90 wt % of cell dry weight; therefore, cells with higher PHB content are clustered on the extreme positive PC-1 axis. These findings show the ability of O-PTIR to identify PHB-producing cells within microbial populations as well as to probe variations in the PHB content within different individual bacterial cells.

**Figure 3:**
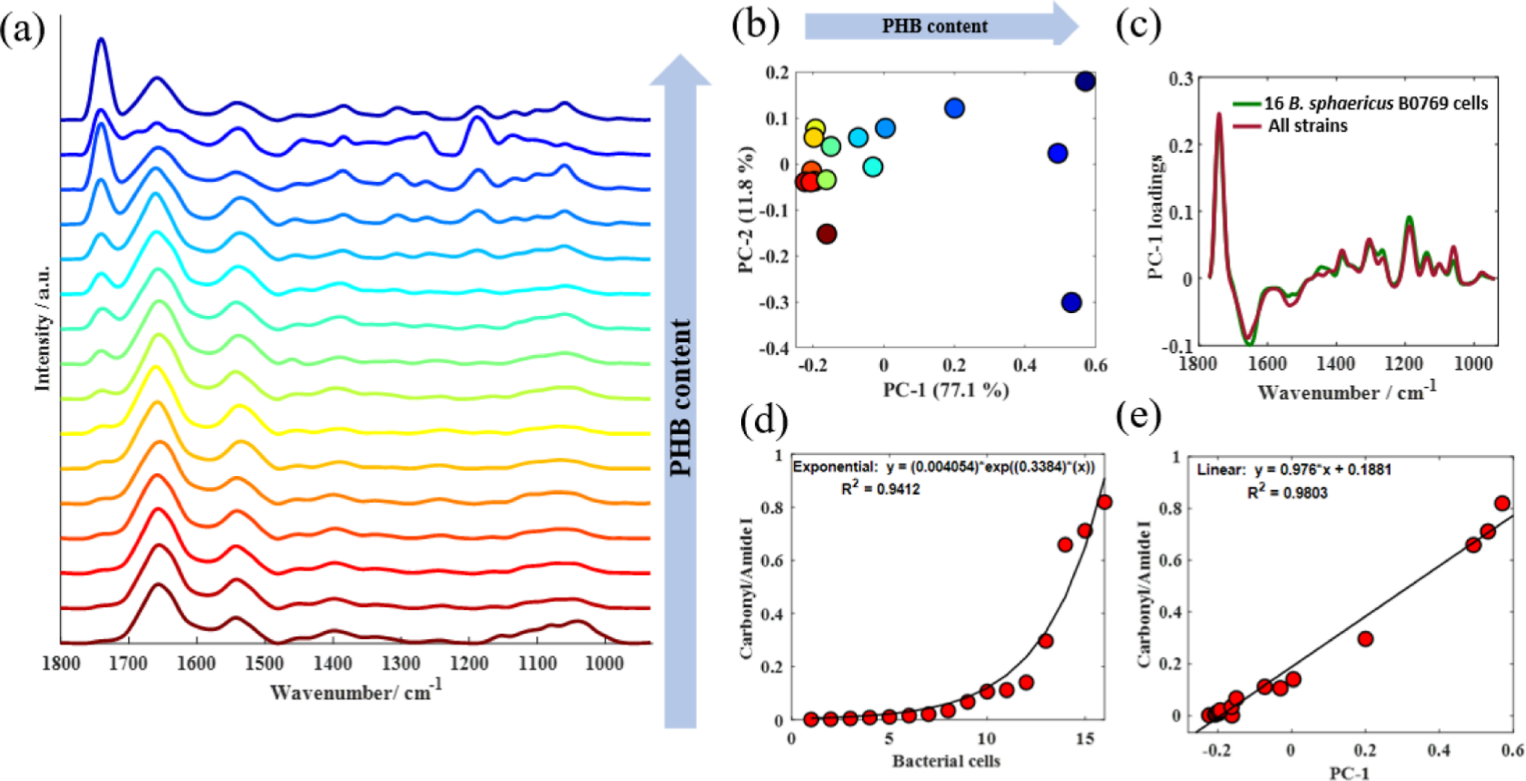
O-PTIR reveals variations in the intracellular PHB content between *B. sphaericus* B0769 cells. (a) O-PTIR spectra acquired from 16 individual *B. sphaericus* B0769 cells from within the same population. (b) PCA score plot obtained from these O-PTIR spectra; values in parentheses represent the percentage of the total explained variance (TEV). (c) PC-1 loading plots obtained from O-PTIR data acquired from 16 *B. sphaericus* B0769 cells (green) compared to PC-1 loadings calculated from input data containing O-PTIR spectra from all 14 Bacillus strains (red). (d) Ester carbonyl/amide I area ratio calculated from infrared signatures from *B. sphaericus* B0769 cells. (e) Correlation between ester carbonyl/amide I area ratio and PC-1. Plots in (a) are offset for clarity and arranged in increasing PHB content from the 1727 cm^–1^/1656 cm^–1^ area ratio.

When focusing on a single PHB-producing strain of *Bacillus*, similar findings were also observed in the infrared signatures of these individual bacterial cells. Figure 3a displays the O-PTIR spectra acquired from 16 *B. sphaericus* B0769 (PHB-positive strain) cells with varying intracellular PHB content. Figure 3b,c illustrates PCA results obtained from O-PTIR data collected from all 16 cells. PC-1 loadings (Figure 3c, green line) are dominated by PHB signatures, and thus, we conclude that the clustering patterns illustrated in the score plots are associated with variations in the intracellular PHB content in *B. sphaericus* B0769 cells. These findings are similar to the results obtained from the analysis comparing all strains (Figure 3c, red line) and were also observed in the PC-1 loadings obtained from the O-PTIR spectra measured from individual bacterial cells within the remaining PHB-producing strains (Figure S1a; Supporting Information). By contrast, PHB signatures were not observed in the PC-1 loadings calculated from infrared data sets acquired from individual bacterial cells from PHB-negative strains (Figure S1b; Supporting Information). These findings show the capability of O-PTIR spectroscopy to monitor variations in intracellular PHB within the same microbial population in a semiquantitative manner. The relative amount of PHB can also be measured by calculating the peak intensity/area of the ester carbonyl band around 1727 cm^–1^ of spectra normalized to the amide I/II band.^36^Figure 3d illustrates the ester carbonyl/amide I area ratio obtained for the infrared signatures from each *B. sphaericus* B0769 bacterial cell, while Figure 3e shows a high correlation between the ester carbonyl/amide I area and PC-1. Thus, with careful calibration, O-PTIR spectroscopy could be used as a tool to quantify intracellular PHB. Previous studies have shown that the PHA content can be quantified from bulk populations using FTIR spectroscopy.^33,36^ In such situations, a calibration curve is constructed based on the FTIR signatures collected from bacterial cultures with varying PHA content plotted against the PHA concentrations measured by another analytical method.^33^ Once calibrated, the method can then be used to predict the concentration of biopolymer from unknown samples in a rapid way.^33,38^ Thus, in order to use an O-PTIR to quantify PHB within individual bacterial cells, a parallel method is necessary to construct a calibration curve. To the best of our knowledge, the only method capable of quantifying bacterial PHB at the single-cell level so far is an approach based on the laser-induced radio frequency plasma charge detection quadrupole ion trap mass spectrometer (LIRFP CD QIT-MS) recently reported by Liang et al.^23^ Further investigations are necessary in order to assess the viability of integrating the O-PTIR platform with LIRFP CD QIT-MS aiming to quantify PHB within a single bacterium; however, the ester carbonyl/amide I ratio can still be used as a semiquantitative method to assess PHB within a single bacterium.

The higher data reproducibility observed in the PCA score plots obtained from FTIR data (Figure 2a) is associated with the lack of sensitivity of FTIR spectroscopy to probe the phenotypic heterogeneities in microbial populations observed at the single-cell level. This is expected as a single FTIR spectrum obtained from bulk populations contains information from thousands of individual bacterial cells that are probed in one single FTIR measurement, which averages out information from subpopulations that are relatively small in number compared to the overall microbial community. By contrast, the infrared signatures retrieved in an O-PTIR spectrum are obtained from a single bacterium. These variations in data reproducibility can also be observed in the averaged spectra and standard deviations calculated from FTIR and O-PTIR data sets (Figure S2; Supporting Information), where PHB-producing strains (*B. subtilis* B0098, *B. sphaericus* B0769, and the two strains of *B. cereus*, *B. megaterium*, and *B. laterosporus*) are easily identified by PHB-related signatures in the spectra. The high standard deviation calculated from the O-PTIR spectra collected from single bacterial cells producing PHB is associated with variations in the PHB content among individual cells within the same bacterial strain.

### O-PTIR Imaging Further Reveals PHB-Producing Cells within Heterogeneous Bacterial Populations

In O-PTIR spectroscopy, a pulsed mid-IR QCL is used to induce photothermal effects on the sample, whereas a visible continuous-wave laser is employed to detect the photothermal effects induced by the QCL. This unique pump-and-probe architecture enables the collection of single-point spectra and chemical maps with high spatial resolution (∼500 nm when a 532 nm laser is used as the probe beam) compared to a standard FTIR microspectrometer (diffraction limited spatial resolution of ∼10 μm at 1000 cm^–1^). The poor spatial resolution in state-of-the-art Fourier transform IR spectrometers is the main reason that most research to date using infrared spectroscopy has focused on analyzing bulk bacterial populations instead of individual cells (typical dimensions of an individual bacterium range from 1 to 2 μm, and these weigh a mere ∼1 pg). A few studies have examined individual bacterial cells by using infrared spectroscopy combined with atomic force microscopy (AFM-IR);^25,26^ however, despite the nanoscale resolution achieved by this platform, near-field optical methods present several challenges such as high cost, complex instrumentation, and relatively long acquisition time and require good contact between the sample and instrument probe.^28^ By contrast, O-PTIR spectroscopy is a far-field technique with ideal resolution to probe single microorganisms as a whole-organism fingerprinting tool, in a contactless manner. In this study, we used O-PTIR imaging to visualize bacterial cell morphology in PHB-producing microbial communities as well as to probe the relative amount of intracellular PHB in bacterial cells. The findings discussed in this section were obtained from *B. sphaericus* B0769 cells, but a similar methodology can be applied to study other bacterial strains.

**Figure 4:**
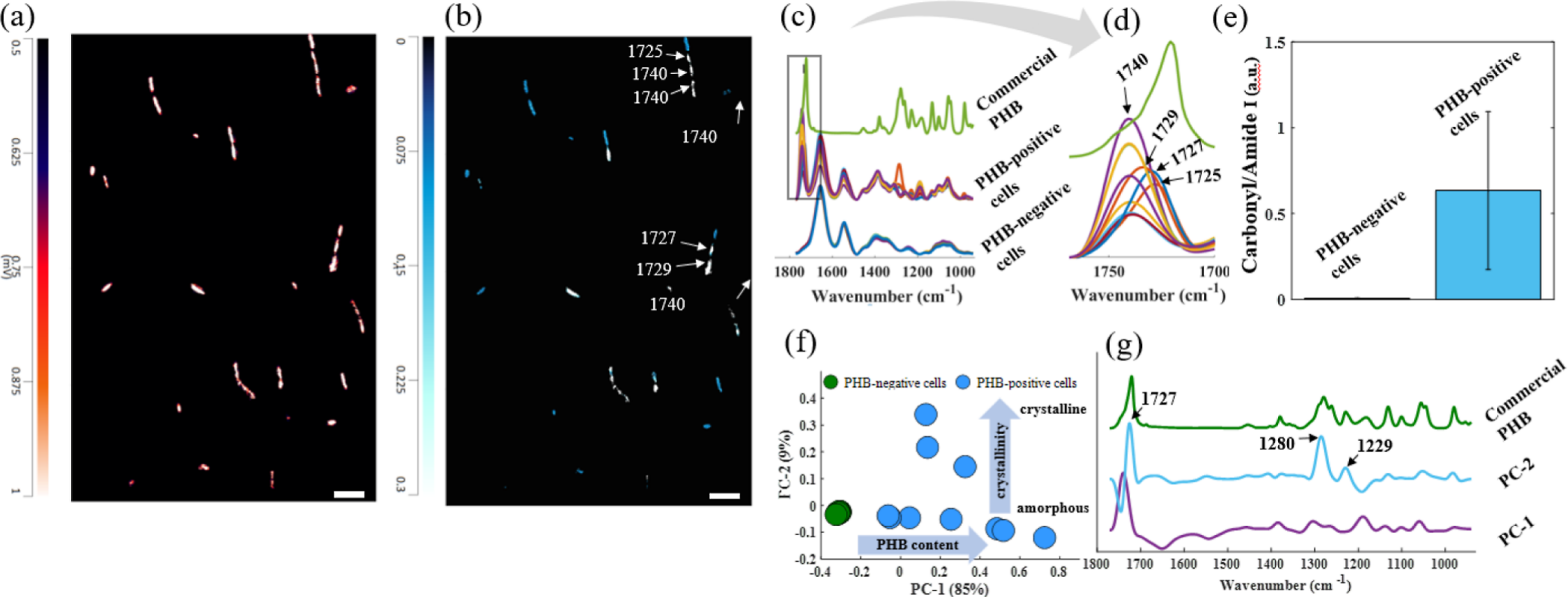
O-PTIR imaging unveils PHB-producing cells within a *B. sphaericus* B0769 population. (a) Single-frequency image showing the distribution of amide I vibration from proteins (1656 cm^–1^). (b) False-color map obtained by the ratio 1727/1656 cm^–1^ showing the relative intracellular PHB content. (c) FTIR spectrum from commercial PHB (green, top) as well as O-PTIR signatures acquired on single-point mode from 18 bacterial cells including eight PHB-negative cells and 10 PHB-positive cells. (d) Details on shifts in the ester carbonyl band stretching vibrations due to variations in PHB crystallinity. (e) Average and standard deviation calculated by the 1727/1656 cm^–1^ area ratio from O-PTIR signatures on single-point mode from cells identified in (b) as being PHB producers (*n* = 10) or nonproducers (*n* = 8). (f,g) PCA scores and loadings plots, respectively, obtained from O-PTIR spectra acquired from 18 bacterial cells; values in parentheses are the percentage total explained variance (TEV). Plots in (c,d,g) are offset for clarity. Scale bar in (a,b) is 8 μm.

Figure 4a shows a single-frequency image obtained by tuning the QCL to amide I vibration from proteins (C=O at 1656 cm^–1^) and the entire bacterial cells can be seen, as these molecules are distributed throughout the whole cell. Imaging the distribution of amide I vibration is commonly used as standard method to identify the exact location of bacterial cells within a sample. Multiple cell arrangements can be identified in Figure 4a, including single rods and chains of varying lengths. It is important to point out that these morphological features may not represent the native morphology of the microbial population, as the sample preparation protocol used in our study may have induced changes in the morphology of the native population due to using deionized water for harvesting and washing, along with the centrifugation steps employed. However, our findings show the ability of O-PTIR imaging to provide morpho-chemical information (i.e., chemical maps containing morphological information) from microbial populations. Figure 4b illustrates a false-color map showing the relative PHB content among cells, which was obtained by calculating the ratio between the maps collected at 1741 and 1656 cm^–1^ (1741/1656). PHB-positive cells exhibit a higher 1741/1656 cm^–1^ index due to the strong contribution of PHB at 1741 cm^–1^; therefore, PHB-richer areas are represented by white pixels, whereas blue pixels are associated with regions with no PHB. O-PTIR signatures acquired in single-point mode from 18 bacterial cells were assessed (Figure 4c) in order to validate the information displayed in Figure 4b regarding the PHB content within bacterial cells. PHB-positive cells showed a higher relative PHB content (1741/1656 cm^–1^ ratio, Figure 4e) compared to PHB-negative cells. PHB content obtained *via* spectral signatures collected on single-point mode were positively correlated with the findings obtained on imaging mode, therefore, indicating the ability of O-PTIR chemical maps to identify PHB-producing cells within microbial populations on imaging mode.

In contrast to the PHB-negative cells, the relative PHB content obtained from PHB-positive cells illustrated a high standard deviation (Figure 4e), suggesting variations in the PHB content among the probed bacterial cells. In order to investigate these variations, PCA was applied to O-PTIR spectra acquired from the 18 bacterial cells on single-point mode. PCA scores (Figure 4f) obtained from the O-PTIR spectra acquired from PHB-negative cells were grouped on the negative axis of PC-1, while scores from PHB-positive cells lay on the positive side of the PC-1 axis with lower data reproducibility compared to the results obtained for PHB-negative cells. Inspection of the PC-1 loadings plots (Figure 4g, purple line) shows that the clustering pattern illustrated on the PC-1 axis is indeed due to varying concentrations of PHB. Interestingly, three PHB-positive cells were grouped on the positive axis of PC-2, whereas scores from the remaining seven PHB-positive cells lay on the PC-2 negative axis in a clear trend along the PC-1 axis (from zero to positive scores). Upon closer examination of PC-2 loadings, PHB-positive cells clustered on the positive axis illustrated positive loadings for bands peaking at 1727, 1280, and 1229 cm^–1^, which have been identified by Porter and Yu as indicators of crystalline PHB.^35^*In vivo*, PHB is found in the bacterial cell cytoplasm as hydrated amorphous granules covered with a monolayer of phospholipids and proteins. The transition of PHB granules from the amorphous to the crystalline state occurs due to changes in the environment surrounding these granules that affect the stabilizers responsible for the amorphousness of native PHB (i.e., lipids, proteins, and water).^35^ The differences observed in the infrared signatures of PHB during crystallization are mainly associated with changes in the intragranular water content, which affects the hydrogen bonding interactions in the polymer in different phases.^34,35^ The ester carbonyl band stretching vibration from PHB is found to have a peak center between 1720 and 1740 cm^–1^ according to the crystallinity state of the molecule. In the crystalline phase, hydrogen bonds are formed between the oxygen atoms from the carbonyl group with hydrogen atoms of surrounding molecules, resulting in a carbonyl vibrational mode peaking toward lower wavenumbers (→1720 cm^–1^).^34^ By contrast, the lack of ordered structure in the amorphous phase leads to reduced hydrogen-bonding effects, thus increasing the wavenumber of absorbance of the ester carbonyl vibration (→1740 cm^–1^). Inspection of the infrared signatures from PHB-positive bacterial cells discussed in Figure 4 reveals distinct states of crystallinity based on the position of the ester carbonyl band stretching vibrations (Figure 4d) in the spectra. PHB-positive cells that illustrated the carbonyl band at 1740 cm^–1^ (amorphous state) resulted in PCA scores clustered on the negative side of PC-2 in a clear trend along the PC-1 axis, whereas spectra from the three remaining cells (ester carbonyl band peaking at: 1725, 1727, and 1729 cm^–1^) generated PCA scores grouped on the positive PC-2 axis. Moreover, the band at 1229 cm^–1^ is associated with the asymmetric stretching of the C–C–O bond due to the formation of helical chains in the crystalline phase, while the band at 1280 cm^–1^ is associated with the CH_2_ wagging vibrations from the C–C–O backbone in the crystalline phase of PHB.^35^ As previously mentioned, the status of intracellular PHB (amorphous or crystalline) depends on the biochemistry of the granule (i.e., lipids, proteins, and water content) as well as how the granule is affected by changes in the environment. Thus, the differences in PHB crystallinity observed in bacterial cells shown in Figure 4 indicate the differences in the intragranular biochemistry of the polymer produced within each cell. The three bacterial cells with crystalline PHB were found arranged in chains as illustrated in Figure 4b according to their ester carbonyl peak vibration. Interestingly, the edges of both bacterial chains are formed by a PHB-producing cell with amorphous PHB at one of the edges and a cell with no PHB in the opposite edge of the chain. These findings could be indicative of a pattern in the intragranular biochemistry of PHB within bacteria arranged in chains. In order to investigate this, additional chains of bacteria were assessed (data not shown). Overall, the vast majority of PHB-producing cells in the evaluated chains illustrated infrared signatures related to amorphous PHB. Individual cells with crystalline PHB were observed on a few chains arranged at random spots on the chain, and cells with no PHB could also be identified in a few cases. These findings indicate no obvious correlation between the arrangements of cells in chains with the crystallinity of intracellular PHB, and therefore the trend observed on the chains of bacteria illustrated in Figure 4 represents an apparent random event (or at least an observation that we cannot explain yet).

According to the findings discussed in this section, a significant variability can be observed in the PHB content within *B. sphaericus* B0769 cells, ranging from whether or not a cell produces PHB to variations in the intragranular biochemistry of PHB within bacterial cells. The presence of subpopulations expressing various phenotypic traits within a microbial community is an evolutionary adaptation strategy called bet-hedging used by bacteria for population growth and survival in an unpredictably changing environment.^15,18,19^ In this hedging strategy, isogenic populations randomly diversify their phenotypes to ensure survival in the face of environmental uncertainty.^14,15,19^ From a bioprocess perspective, phenotypic heterogeneity among microbial cell factories may negatively affect bioprocesses as the overall productivity of the system can be affected by the growth of subpopulations with inefficient producer cells.^12^ Therefore, monitoring the dynamic of bacterial populations undergoing bet-hedging and understanding their mechanisms for dealing with environmental stress are critical for optimizing the robustness of bioprocesses. Here, we demonstrate the ability of O-PTIR spectroscopy to probe phenotypic heterogeneity among individual bacterial cells within microbial populations producing PHB; therefore, the method is a promising tool for monitoring microbial bioprocesses for PHB production. Although O-PTIR measurements discussed here were acquired from dry samples, which might be an issue for online monitoring of bioprocesses, previous studies have shown that infrared measurements acquired through mid-infrared absorption-induced photothermal effect can also be performed on living cells in aqueous samples.^39,40^ More recently, Yin *et al.* reported a novel imaging technique operating with similar working principles of O-PTIR imaging that enables to acquire infrared chemical maps from aqueous solutions at video-rate speed, which opens up opportunities to monitor the dynamics of biological processes in real time.^40^ Ultimately, we highlight that O-PTIR spectroscopy can also be coupled with fluorescence and Raman spectroscopies to acquire spectral data from the same sample location at the same high spatial resolution,^30,31,41^ expanding the range of applications of this emerging technique to various fields of research including characterization of materials, online measurements at the industrial scale, as well as *in vivo* measurements.

## Conclusions

In summary, our study shows that O-PTIR spectroscopy can monitor phenotype heterogeneity within *Bacillus* populations producing PHB. Measurements obtained on single-point mode can be used as a rapid tool to assess PHB content within bacterial cells in a semiquantitative manner as well as to probe variations in the intragranular biochemistry of PHB. Infrared chemical maps acquired in imaging mode provide insights into the spatial distribution and arrangement of PHB-producing cells within a microbial population. The ability of O-PTIR spectroscopy to monitor changes in the bacterial phenotype at the single-cell level as a whole-organism fingerprinting method opens up further opportunities in single-cell microbial metabolomics such as evolution of microbial cells, development of microbial antibiotic resistance, quorum sensing, and the interaction of bacteria with host immune cells.

## Supporting Information Available

Bacterial strains and growth conditions, data analysis, PC-1 loading plots obtained by subjecting O-PTIR spectra acquired from individual bacterial cells from each strain, and mean and standard deviations calculated from FTIR and O-PTIR spectral data sets (PDF)
