# Supplementary material for: Monitoring Phenotype Heterogeneity at the Single-Cell Level within Bacillus Populations Producing Poly-3-hydroxybutyrate by Label-Free Super-resolution Infrared Imaging
Source: Anal Chem. 2023 Nov 24;95(48):17733–40. doi: 10.1021/acs.analchem.3c03595 (PMC10701708; doi:10.1021/acs.analchem.3c03595)
Supplement: Supplementary file 1 — ac3c03595_si_001.pdf [file ac3c03595_si_001.pdf]

Supporting information

Monitoring phenotype heterogeneity at the single cell level within *Bacillus* populations producing poly-3-hydroxybutyrate by label-free super-resolution infrared imaging

Cassio Lima[1], Howbeer Muhamadali[1], and Royston Goodacre[1, *]

[1] Centre for Metabolomics Research, Department of Biochemistry, Cell and Systems Biology, Institute of Systems, Molecular and Integrative Biology, University of Liverpool, Liverpool L69 7ZB, United Kingdom

Contents

Experimental section – Bacterial strains and growth conditions……..……………………………..….S3
Experimental section – Data analysis……………………………………………………………...S4
Additional results ………………………………………………………………………………S5-S6

I. EXPERIMENTAL SECTION

Bacterial strains and growth conditions

Bacterial strains used in this study consist of 14 *Bacillus* species (Table 1). All bacterial strains were cultured on LB-agar plates at 37 °C for 24 h. After this incubation period, bacterial samples were prepared by harvesting the biomass from the surface of each plate using sterile inoculating loops and re-suspended in 1 mL of deionized water. Samples were centrifuged at room temperature for 4 min at 4000 *g* using a benchtop Eppendorf microcentrifuge 5424R (Eppendorf Ltd., Cambridge, U.K.). The supernatants were discarded and the biomass washed by re-suspending in 1 mL of deionized water, followed by a centrifugation step to remove any residues from the media. Bacterial concentrations were adjusted to an optical density (OD_{600}) of 15.

Table 1. List of *Bacillus* strains examined in this study.

**Bacterial strain**
B. amyloliquefaciens B0177
B. amyloliquefaciens B0620
B. cereus B0002
B. cereus B0550
B. laterosporus B0043
B. laterosporus B0262
B. licheniformis B0242
B. licheniformis B1081
B. megaterium B0076
B. megaterium B0010
B. sphaericus B0769
B. sphaericus B7134
B. subtilis B0014
B. subtilis B0098

Data analysis

The baseline of spectral data was corrected using an algorithm based on asymmetric least squares, while a Savitzky−Golay filter (polynomial of second order in a 7-point window) was used as smoothing filter. All spectra were vector normalized prior to statistical analysis. All statistical analysis was carried out in MATLAB version 2022a (The Mathworks Inc., Natwick, US) using code found in our GitHub repository (https://github.com/Biospec/).

II. RESULTS AND DISCUSSION

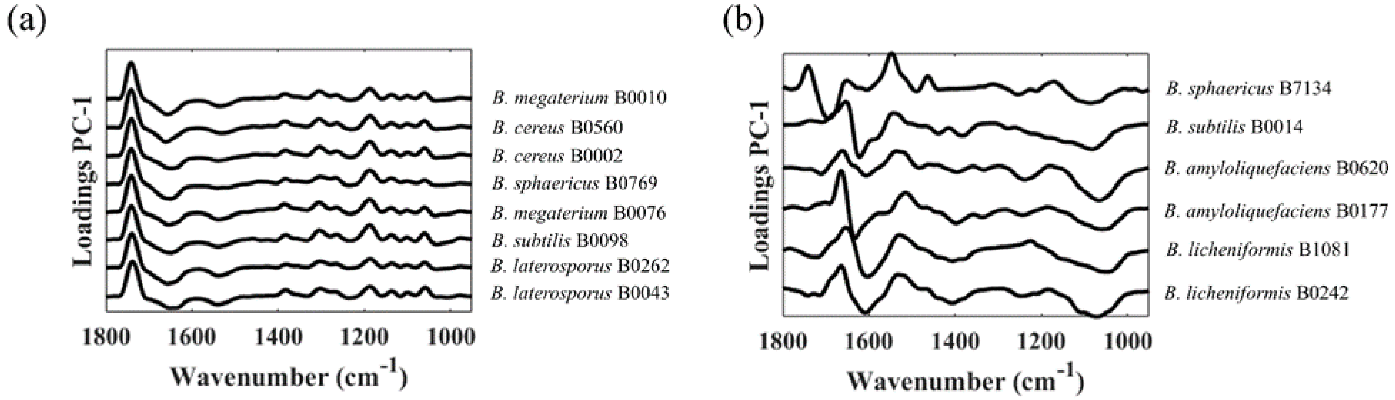

Figure S1. PC-1 loadings plots obtained by subjecting O-PTIR spectra acquired from individual bacterial cells from each PHB-positive (A) and PHB-negative strain (B). Plots are offset for clarity.

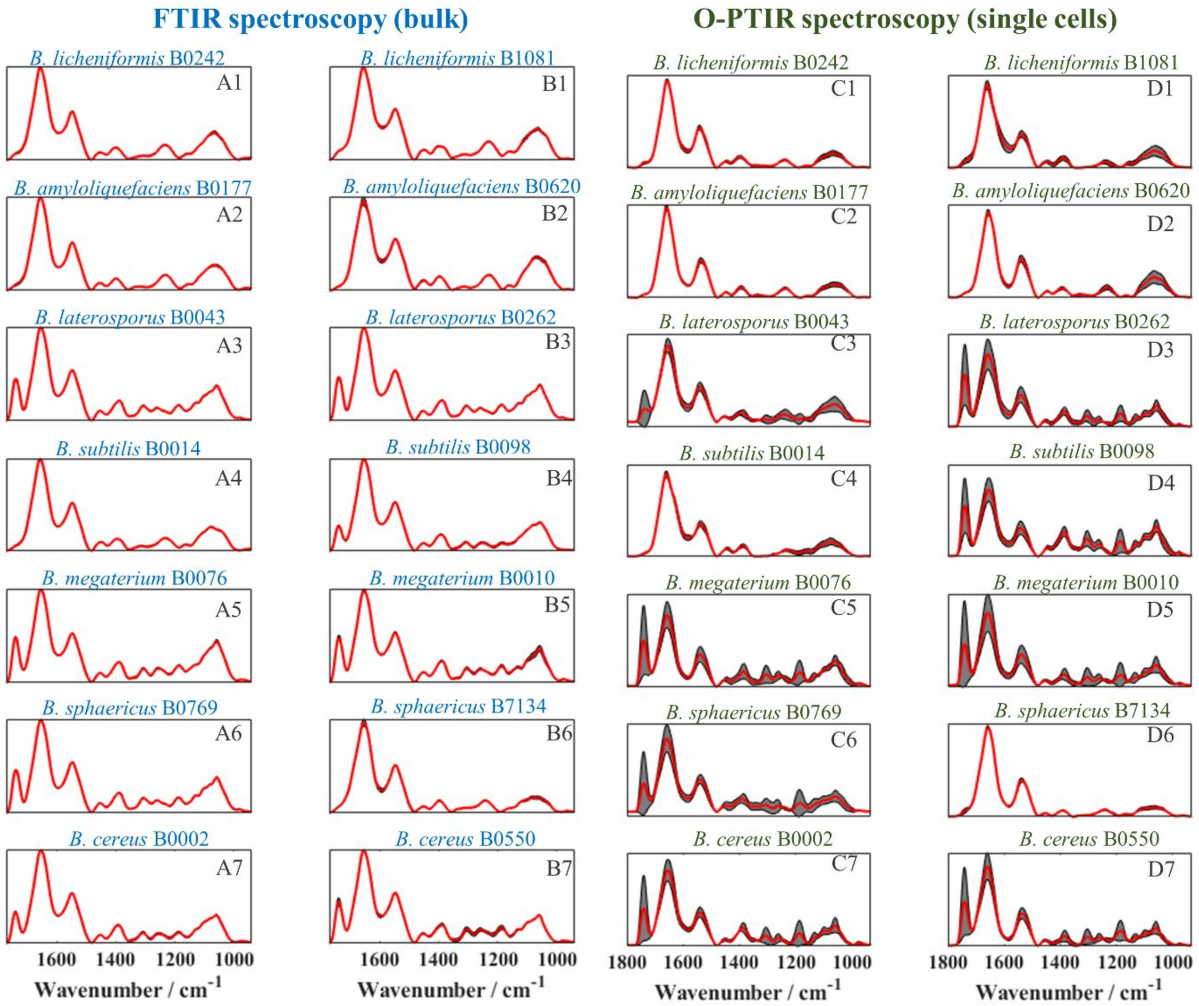

Figure S2. Mean and standard deviations calculated from FTIR spectra collected from bulk (panels A and B) and O-PTIR spectra acquired from single bacterial cells (panels C and D) from all fourteen strains used in this study. For FTIR 16 repeats were taken and for O-PTIR 10 individual cells were analyzed.
